# Functional MRI of the Olfactory System in Conscious Dogs

**DOI:** 10.1371/journal.pone.0086362

**Published:** 2014-01-23

**Authors:** Hao Jia, Oleg M. Pustovyy, Paul Waggoner, Ronald J. Beyers, John Schumacher, Chester Wildey, Jay Barrett, Edward Morrison, Nouha Salibi, Thomas S. Denney, Vitaly J. Vodyanoy, Gopikrishna Deshpande

**Affiliations:** 1 MRI Research Center, Department of Electrical & Computer Engineering, Auburn University, Auburn, Alabama, United States of America; 2 Department of Anatomy, Physiology & Pharmacology, Auburn University, Auburn, Alabama, United States of America; 3 Canine Detection Research Institute, Auburn University, Auburn, Alabama, United States of America; 4 Department of Clinical Sciences, Auburn University, Auburn, Alabama, United States of America; 5 MRRA Inc., Euless, Texas, United States of America; 6 College of Veterinary Medicine, Auburn University, Auburn, Alabama, United States of America; 7 MR R&D, Siemens Healthcare, Malvern, Pennsylvania, United States of America; 8 Department of Psychology, Auburn University, Auburn, Alabama, United States of America; CNRS, France

## Abstract

We depend upon the olfactory abilities of dogs for critical tasks such as detecting bombs, landmines, other hazardous chemicals and illicit substances. Hence, a mechanistic understanding of the olfactory system in dogs is of great scientific interest. Previous studies explored this aspect at the cellular and behavior levels; however, the cognitive-level neural substrates linking them have never been explored. This is critical given the fact that behavior is driven by filtered sensory representations in higher order cognitive areas rather than the raw odor maps of the olfactory bulb. Since sedated dogs cannot sniff, we investigated this using functional magnetic resonance imaging of conscious dogs. We addressed the technical challenges of head motion using a two pronged strategy of behavioral training to keep dogs' head as still as possible and a single camera optical head motion tracking system to account for residual jerky movements. We built a custom computer-controlled odorant delivery system which was synchronized with image acquisition, allowing the investigation of brain regions activated by odors. The olfactory bulb and piriform lobes were commonly activated in both awake and anesthetized dogs, while the frontal cortex was activated mainly in conscious dogs. Comparison of responses to low and high odor intensity showed differences in either the strength or spatial extent of activation in the olfactory bulb, piriform lobes, cerebellum, and frontal cortex. Our results demonstrate the viability of the proposed method for functional imaging of the olfactory system in conscious dogs. This could potentially open up a new field of research in detector dog technology.

## Introduction

**Figure 1:**
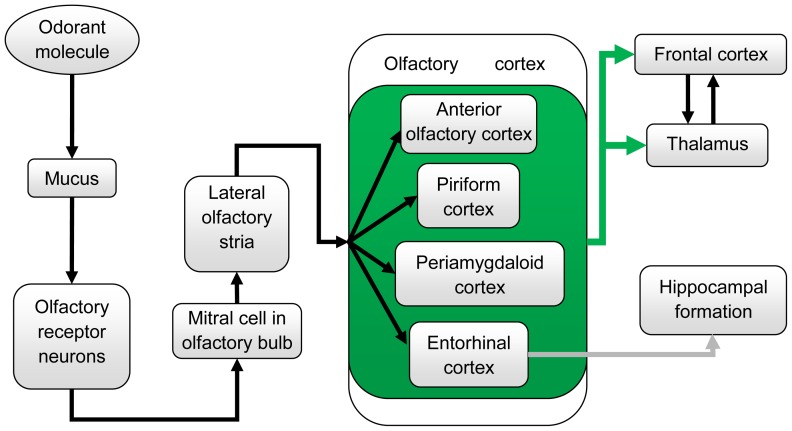
A schematic of the olfactory pathway in canines. Arrows indicate the olfactory signal flow. Anterior olfactory cortex, piriform cortex, periamygdaloid cortex, and entorhinal cortex are contained in a green box and the green arrows extending from this green box indicate the olfactory signal from them go to frontal cortex and thalamus. The gray arrow from entorhinal cortex to hippocampal formation indicates the olfactory signal to hippocampus comes only from the entorhinal cortex. For functions of each site, please refer to [1]–[4], [6], [7].

The properties of the dog's olfactory system result from physical and biochemical events that occur at the olfactory epithelium of its nasal cavity where olfactory receptor neurons interact with odorants. Based on previous reports involving *in vitro* studies and *in vivo* studies in canines and other species (mainly humans), we can construct the following hypothesis about the cerebral architecture of the canine olfactory system. Olfaction begins with sniffing, which transports odorant molecules into the nose and delivers them to the mucus layer covering the olfactory epithelium [1]. The binding of the odorant by a receptor protein initiates an intracellular cascade of signal transduction events, including the G-protein-dependent production of second messenger molecules, leading to opening of ion channels and passing of ion currents. This process triggers an action potential in the axon of the olfactory receptor neuron that projects directly to the olfactory bulb (OB) [1], [2]. The OB generally functions as a filter and has three non-exclusive functions: discriminating among odors, enhancing sensitivity of odor detection and filtering out background odors to enhance the transmission of selected odors. OB neurons then transmit signals to pyramidal neurons in the olfactory cortex that is composed of the anterior olfactory cortex, piriform cortex, periamygdaloid cortex and entorhinal cortex. The anterior olfactory cortex detects and stores correlations between olfactory features, creating representations (gestalts) for particular odorants and odorant mixtures, as shown by Haberly in humans [3]. Piriform cortex carries out functions that detects and learns correlations between olfactory gestalts formed in anterior olfactory cortex and a large repertoire of behavioral, cognitive and contextual information to which it has access through reciprocal connections with frontal, entorhinal, and periamygdaloid areas [3]. The periamygdaloid area participates in emotional processing of olfactory stimuli and facilitates memory encoding, as shown by Zald et al with human PET (positron emission tomography) data [4], and entorhinal cortex functions as a hub for memory network and navigation [3]. The pathway then projects to the hippocampal formation and thalamus, which relays information to neocortical areas such as the medial and orbitofrontal cortex where the olfactory signal is interpreted [5] (see Fig. 1 for schematic). The medial and orbital parts of the frontal cortex are known to be involved in cognitive integration of all sensory stimuli in relation to prior experiences, as shown using functional neuroimaging in humans [6]. The hippocampal formation is involved in recognition memory of odors [3]. The thalamus' involvement in olfaction as a relaying hub is still under debate, however its involvement in odor thresholding has been acknowledged, at least in humans [7]. Also, previous studies have shown that dogs possess much more olfactory receptors per square centimeter of the olfactory epithelium as compared to humans [8]. This clearly demonstrates the dogs' advantage over humans in sensory transduction at the cellular level. However, how this advantage is carried forward higher into the odorant detection chain remains unexplored.

While much is known about the canine olfactory system at the *in vitro* cellular level [9]–[14] and behavioral level [15], [16], little work has been done at the cognitive level, which is an important and largely unexplored link in the series of events leading to odor detection. It is critical to bridge the gap between cellular findings and systemic behavioral observations by investigating the sense of smell at the cognitive level. For example, an increase in the concentration of odorant will induce a change in response at the cellular level according to Weber's law [17]. How this change in response translates to a change in odorant detection *in vivo*, however, is unknown, and could potentially be explored by using functional magnetic resonance imaging (fMRI), which allows noninvasive mapping of brain function without the administration of an exogenous contrast agent [18], [19].

In this study, we characterized the cognitive response in dogs' brain to odorants of different concentrations using dogs that were fully conscious (i.e., awake), and compared the response to that of lightly sedated dogs. Awake animal fMRI studies are methodologically challenging but more valuable because the data reflect the brain activity under more ecologically valid conditions. Anesthesia affects the neural activity as well as the regulation of cerebral circulation significantly [20], [21]. Only a small amount of animal fMRI studies have been published under awake conditions due to the difficulty of restricting motion effect [20]–[22]. Here, awake dog imaging was made possible by using an optical head motion tracking system [23] for retrospectively correcting for artifacts created by head motion. FMRI provides for the apparatus to investigate the dog olfactory system *in vivo* and noninvasively, and has been used to study the neural basis of olfaction in humans [24], [25], monkeys [26] and rodents [27]. But, to our knowledge, there has been no investigation of the olfactory system in dogs (either awake or sedated) using fMRI or any other imaging modality. Dog imaging techniques developed in this study measure activity in the olfaction-related brain areas and quantify concomitant changes with different odorant concentrations. We believe that the techniques we report here will serve as a seminal noninvasive method for the exploration of the dog's olfactory system at the cognitive level.

The broader impacts of this endeavor are multifold. Currently, we depend upon the olfactory abilities of dogs for what are considered highly specialized and critical tasks such as detecting explosive devices, hazardous chemicals, and illicit substances. The societal importance of such tasks has increased efforts to enhance the technological sophistication by which canine olfaction is employed. To support such enhancement, greater emphasis has been placed on understanding fundamental olfactory function and capacities. Non-invasive imaging techniques such as fMRI, coupled with animal psychophysics based techniques, hold significant promise for advancing the understanding of fundamental olfactory function and associated cognitive processing of odor sensory information. A mechanistic understanding of canine odorant detection is not only important from a basic science perspective but also to understand how canine detection capabilities change with different odorants and operating environments. Federal agencies in United States (Federal Aviation Administration, Department of Homeland Security and Department of Defense) have invested millions of dollars to support research into basic science of canine odor detection and training because of the increase in terrorism threats, domestic and foreign narcotics trafficking.

## Material and Methods

### Ethics Statement

Approval was obtained from the Auburn University Institutional Animal Care and Use Committee for performing this study.

### Overview of the Dog fMRI Olfactory Imaging System

**Figure 2:**
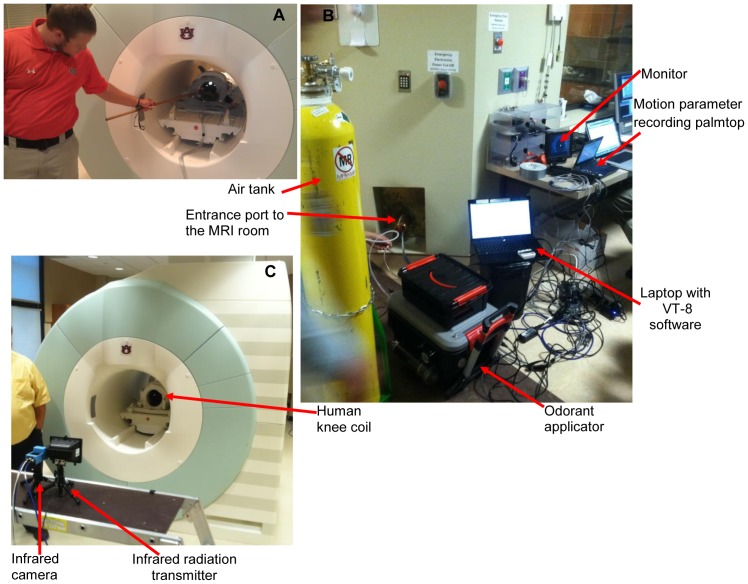
Components of the dog fMRI olfactory imaging system. (A) dog training to insert and keep their heads as still as possible inside the human knee coil using positive reinforcement learning (a black dog can be seen inside the coil); (B) components of imaging system outside the MRI room showing odorant applicator, air tank, motion parameter recording palmtop, video monitor, laptop with VT-8 software (see explanation in “Methods and Material: olfactory stimulus device”), and the entrance port to the MRI room; (C) components of imaging system inside the MRI room, showing human knee coil, infrared camera and infrared radiator.

**Figure 3:**
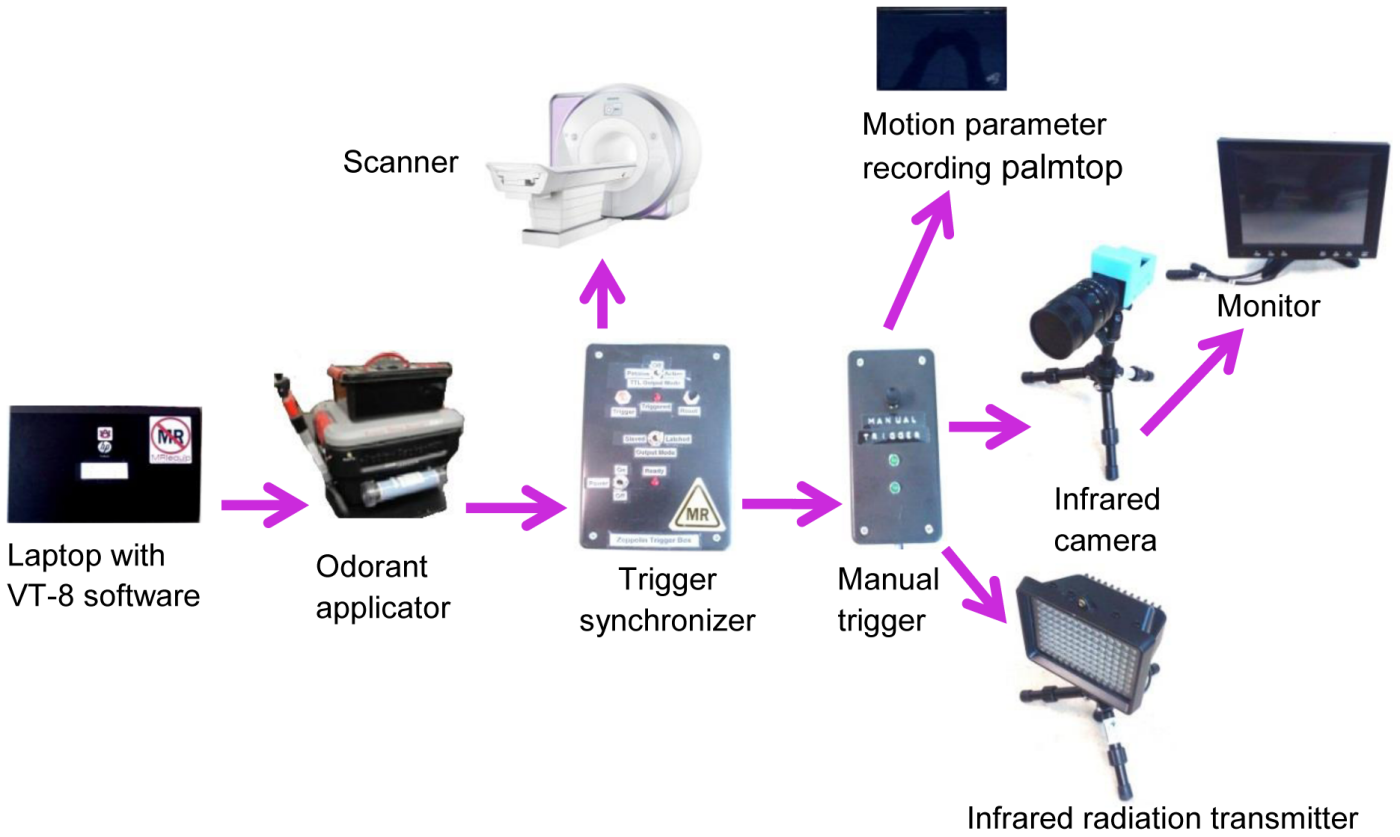
The interlinked trigger system. Arrows denote the triggering direction. A laptop with VT-8 software [35] provided the interface to trigger the odorant applicator. The VT-8 software is a platform that can be used to design and display sequence of odorant flow and clearance, and provides communication and control to odorant applicator to generate the expected experimental sequence. Once the odorant applicator started to give odorant stimulus, it sent a signal to the trigger synchronizer, which then triggered the scanner and sent a signal to the manual trigger. The manual trigger, as the name suggests, was manually set for switching between two states. One was waiting for signals from trigger synchronizer, and the other was waiting for signal from a hand-pressed button. In our experiment, the first state was used for data collection and the second was used only for testing. Upon receiving the signal, it triggered the infrared radiation transmitter to give off infrared rays, and the infrared camera to start recording infrared reflections from the dog's head, and the motion parameter recording palmtop to start calculating displacement parameters. When the camera was triggered, it sent the signal to the monitor for display.

The components of the dog fMRI olfactory imaging system are shown in Fig. 2. First, the dogs were trained to insert and keep their heads as still as possible inside the human knee coil when being scanned (Fig. 2(A)). This was achieved by positive reinforcement training techniques using a target stick and bridging stimulus (i.e., clicker) for head placement maintained by delivery of edible treats for emission of desired responses. Second, a custom-built odor applicator was used for controlled delivery of odorant stimulus (Fig. 2(B)). Third, the scanner system consisted of a 3T Siemens Verio scanner and a human knee coil which perfectly fit into the role of a dog head coil. Fourth, an optical head motion tracking system was employed for tracking dog head motion during fMRI (Fig. 2(B), (C)), and consisted of an infrared (IR) camera, an IR illuminator, a video monitor, and a data recording palmtop. We declare that the person in the photograph has given written informed consent, as outlined in the PLOS consent form, to publication of their photograph. A schematic of the interlinking and triggering among them is shown in Fig. 3. Finally, post-processing of functional data was performed with SPM8 (statistical parametric mapping) [28].

### Dog Training and Preparation

We recruited six Labrador Retriever dogs (five males) from the Auburn University Canine Detection Research Institute with ages in the range of 12 to 60 months. For anesthetized imaging, dogs were sedated with intramuscularly administered xylazine (2.2 mg/kg) and lightly anesthetized with ketamine HCl (11 mg/kg). For awake imaging, the dogs were trained to move to the correct position within the scanner, insert their heads within a human knee coil, and remain still for the required duration of imaging using positive reinforcement behavior shaping procedures.

All training used positive reinforcement to obtain the desired performance by the dogs. Prior to training for fMRI scanning, the dogs were trained to follow, touch, and remain touching the end of a “target-stick” (3/8 inch diameter, 36 inch long, wooden dowel with red tape as target on one end) with their nose using small bits of commercial dog food treats as rewards to shape and maintain this response. Concomitantly, a tin clicker was established as a conditioned reinforcer by pairing the click with the delivery of food treats for correctly touching and holding their nose to a touch-stick. Such a conditioned reinforcer is also known as a “bridge” because it provides an immediate signal to the animal that the desired response has been emitted and bridges the gap in time until a food treat can be delivered. This sort of target-stick and bridging signal training is a common practice in pet animal as well as professional husbandry with animals in zoos to train them to present themselves for medical monitoring and treatment, conduct educational/entertainment animal shows, and to generally reduce the dangers associated with managing animals. The use of the clicker allows more precise delivery of a rewarding stimulus to shape (i.e., build, develop) a desired response. Establishing the target-stick repertoire to a proficiency level adequate for moving onto training for fMRI took from 30 minutes to an hour for each dog.

The first round of training for awake, unrestrained fMRI of the dogs took place outside of the actual scanning using a fixture to replicate the human knee coil (i.e., 2.5 gal plastic bucket with the bottom cut out) affixed to one end of a table that approximated the height and width of the MRI (magnetic resonance imaging) table. The dogs were prompted to jump up on the table or, if unable to easily jump on the table, place their front paws upon the edge of the table so they could be easily lifted upon the table by one person. The previously established clicker and target-stick repertoire was used to train the dog to place its head within the simulated MRI coil and position its nose within the olfactory stimulus delivery mask, which was affixed inside the simulated human knee coil. The dogs were trained to hold their heads relatively motionless within the simulated coil by clicking the clicker only when their head was in the correct position and held still. The amount of time that a dog had to hold its head still in the correct position to receive the click followed by a food treat was gradually increased. Meanwhile, throughout this process, a recording of the sound from the operation of the MRI was played through a portable stereo, the volume of which was gradually increased until similar in intensity to that of being in the actual scanning. The final training performance was the dog holding its head relatively motionless in the correct position while the MRI sounds were played at approximately the same intensity as that of being in the actual MRI for 5 minutes and repeating this performance several times across the course of an hour-long training session. This training phase took, on average, about 20, 1-hour training sessions across a 4-week period for each dog.

The second round of training was performed inside the real scanner with the human knee coil and with running of the functional and structural sequences. The dogs were acclimated and transitioned to performing the head positioning response in the actual MRI scanner in one, approximately hour-long session each. The dog trainer always accompanied and monitored it (see Fig. 2(A)) in the scanning room. The dog was prompted onto the MRI table, into the MRI core, to place its head within the human knee coil, and position its nose in the olfactory stimulus delivery mask. Starting with a relatively short duration of holding its head in position and relatively motionless with the fMRI intermittently operating, the time requirement for receiving a click followed by a food treat was variably and rapidly increased until the dog reliably executed the performance for one fMRI sequence to be used in the experiment. The click was presented at the end of the sequence followed by delivery of the food treat from the hand of the trainer to the dog. A recent paper demonstrated a similar training approach for imaging the reward system in awake dogs [29]. However, even with training, some head movement was inevitable (e.g. respiratory repositioning). Therefore, we used a single camera optical head motion tracker to monitor the motion of the dog's head and retrospectively correct for motion effect.

### Olfactory Stimulus Device

#### Odorant Delivery

The accurate delivery of odor stimulus is very important in olfactory physiological experiments. When used with fMRI, demanding additional constraints are placed on olfactometers [30]–[33]. The most obvious one is the absence of any magnetized material in the MRI room. Other features of the instrument include computer control and odorant presentation of accurate and reproducible duration of a preselected sequence with no additional stimulation (e.g., tactile, auditory) [30], [34].

**Figure 4:**
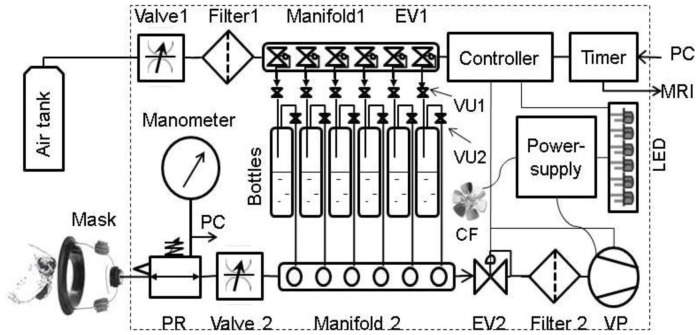
Diagrammatic representation of the olfactory stimulation device. The device consists of inflow & clearance air paths and an electronic control system. The inflow air/odorant path consists of a tank, flow control Valve 1, dry-rite type air Filter 1, Manifold 1 (6 isolated channels with electronically controlled valves), an electronic valve (EV1), 6 unidirectional pressure controlled valves (VU1, VU2), 6 odor bottles, Manifold 2 (6 flow-through isolated channels), flow control Valve 2, pressure regulated valve (PR), and electronic Manometer. The clearance path includes vacuum pump (VP), charcoal Filter 2, and electronic valve (EV2). The electronic control system consists of a 6-channel valve Timer, 6-channel valve Controller; Power-supply (feeds the VP, the visual LED control pane, and the cooling fan (CF)). Power for the Timer and Controller comes from the personal computer (PC). The protocols of timing and sequencing are stored and directed by the PC connected to the Timer. The Timer is synchronized with MRI. The experimental air pressure directed to the Mask are measured by the electronic Manometer and recorded by PC.

We built a custom device for the precise computer-controlled delivery of pre-determined quantities of odorants over a precise time interval (Fig. 4). The device provided for the flow of air under pressure through a series of filters, valves, and manifolds to sweep the headspace over containers into a mask, for the precise quantitative delivery of odorants to the nasal cavity of dogs. A vacuum suction then cleared the odorant after a precise amount of time. In this manner, the device controlled the precise extent and time of exposure of substances to olfactory tissue. The moisture content of the air was controlled to a constant humidity using a drierite type air filter. The drierite type air filter is a purifier that is specially designed for gas chromatography and other applications requiring pure and dry gas. It dries, purifies, and filters gases used for chromatography and spectrometry.

During the fMRI experiment, the air tank, odorant applicator, and computer were positioned outside the MRI room in the close proximity from the utility entrance port into the MRI room. Six 6-mm plastic tubes were passing through the entrance port for connection with the animal mask. Each tube, channel, and bottle was used only for the particular odorant sample to avoid cross contamination. Valve 2 and pressure regulator (PR) (see Fig. 4) were purged and cleaned after each experimental session. All materials and components used in this device were chemically stable and are not odoriferous.

As illustrated in Fig. 4, the computer (PC) used VT-8 software [35] to send a signal to the VT-8 Warner Valve Timer (indicated as “Timer” in Fig. 4) that in turn communicated with the VC-8 Warner Valve Controller (Controller) to open one of six Oxygen Clean 2-way normally closed electronic valves (EV1) installed in the 6-port Oxygen Clean Manifold (Clippard Instrument Laboratory, Manifold 1). When EV1 was open, the corresponding LED (light emitting diode) control light was on, and air entered from the Air Tank into the Miniature Clippard Air Flow Control Valve (Valve 1). Then through the W.A. Hammond Drierite Laboratory Gas Drying Unit (Filter1) the air went to the open EV1, the first Clippard Unidirectional Valve (VU1), the head space of 100 mL bottle, and then the second Clippard Unidirectional Valve (VU2). After that, it followed through the corresponding normally open channel of the Clippard Manifold 2 and via the second Miniature Clippard Air Flow Control Valve (Valve 2) to the Clippard Pressure Regulator (PR), with air pressure measured by DT-8890CEM Ruby-electronics Digital Differential Air Vapor Pressure Meter Gauge Manometer (Manometer). Finally, the air with odorant exited the odor applicator and entered the SurgiVet Pet Oxygen Mask (Mask) [36] via the 6-mm tubing.

At the end of activation time (10 s) the Controller closed EV1 in the Manifold 1 and simultaneously opened the Oxygen Clean 2-way normally closed electronic valve (EV2) and the vacuum pump (AM6BS Metropolitan Vacuum) (VP) in the applicator exhaust path. The air with odorant was cleared from Mask, Valve 2, the open channel of Manifold 2, EV2, and W.A. Hammond Carbon Filter (Filter 2). After the clearance of odorant (10 s) the EV2 and the vacuum pump were shut off and the system rested 20 s before a new activation began. The full cycle of the odor applicator, therefore, was typically composed of 10 s of odor application and 30 s of no odor. The no odor time was composed of 10 s clearance of odorant and 20 s rest time.

The pressure pulses were measured by Manometer and sent to computer (PC). The odor applicator was synchronized with MRI by the Timer signal sent to MRI computer. A cooling fan (CF) helped to maintain the temperature of odorants and device components. We used a known odorant mixture of ethyl butyrate, eugenol, and (+) and (−) carvone in water at concentrations of 0.016 mM (low concentration) and 0.16 mM (high concentration) each [37].

**Figure 5:**
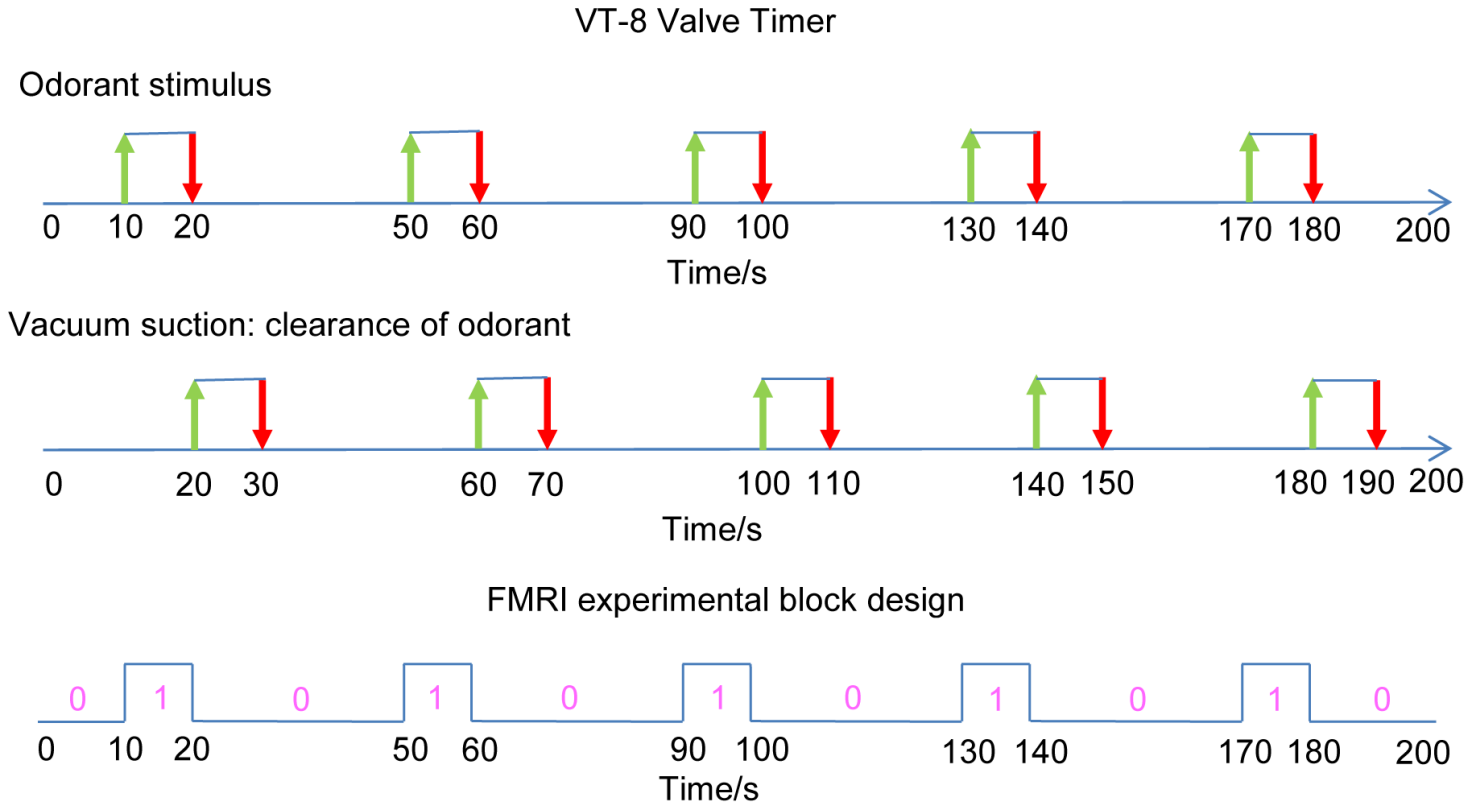
Odorant applicator sequences controlled by VT-8 Warner Timer software and fMRI experimental block design. For the first sequence, green arrows indicate the onset time of the odorant stimulus and red arrows indicate when the stimulation ends. For the second one, green arrows indicate the onset of clearance of odorant, and red arrows indicate when it ends. The third sequence shows the fMRI block design in this work, matching the first sequence. “0” and “1” denote the odor “on” and “off” conditions.

Plastic caps were modified for air-tight connection with the bottles containing odor solutions. The removable platform for bottles containing odorants, LED assembly and various clamps for positioning the parts were custom built. The odor applicator was controlled by the Warner VT-8 software [35] and was programmed to generate the experimental sequence of odorant flow and clearance (Fig. 5). Since the odorant airstream was unwarmed and introduced at room humidity, excessive flow rate was not desirable. Also, animals subjected to continuous un-humidified flows could have nasal drying and discomfort. High air flow can present air flow turbulence, impacting rise times [38]. Because the mask had two valves that supported unrestricted exhaling and inhaling, the incoming air flow was limited to 1 l/min based on American Animal Hospital Association (AAHA) guidelines [38].

#### Mask

**Figure 6:**
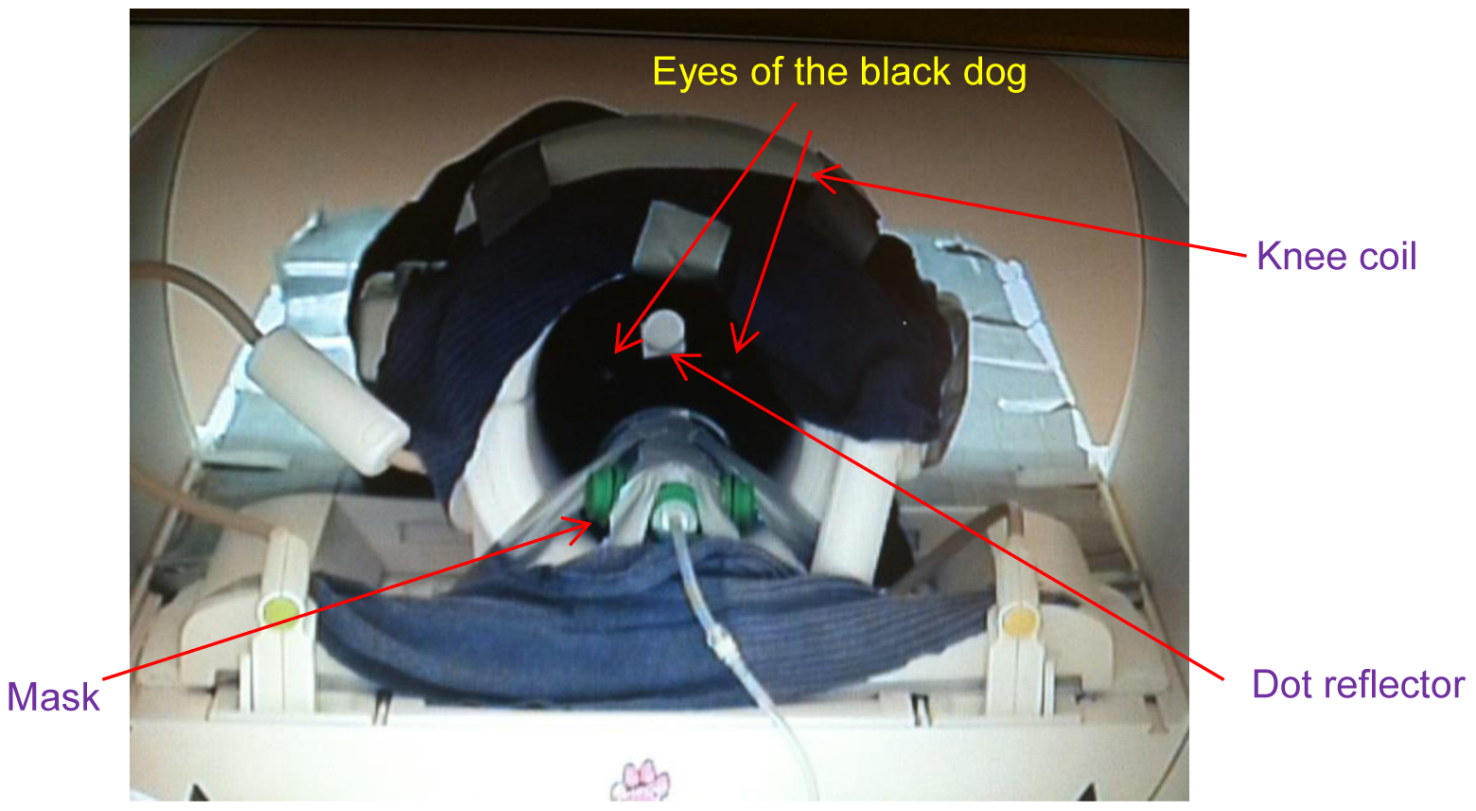
A black dog positioned with muzzle in mask for odorant delivery. The dot reflector is mounted to dog's head for motion tracking, the knee coil encompasses the dog's head and the mask is mounted on the front frame of the knee coil.

A SurgiVet Pet Oxygen Mask [36] was used in our experiments. This mask is made of polycarbonate and has two valves that aid unrestricted exhaling and inhaling, and a port to which the tube for odorant delivery and evacuation was attached. The mask was mounted on the frame of the knee coil such that when the dog placed its nose in the mask, its head was correctly positioned within the coil for imaging (Fig. 6).

### Optical Head Motion Tracking

Head movement is a critical issue for all modalities of brain imaging, especially for fMRI. Excessive movement results in image ghosting and blurring. Because head movement is a significant obstacle in imaging studies of conscious animals, and some cognitive processes such as olfaction are impossible to comprehensively study in anesthetized animals [20]–[22], we have adopted an approach involving both dog training and optical head motion tracking. Even the best trained dogs will inevitably make slight, sometimes jerky, head movement. In the present case, the dogs would sometimes pant, which is a physiologically mediated response that is difficult to reliably control through positive reinforcement procedures. Such motions are difficult to remove using post-hoc image transformations. Therefore, the ability to compensate or reduce motion artifacts is one of the most challenging difficulties while acquiring MR images from a conscious animal. To solve this problem, two approaches have been tried in the past. First, the head of the animal can be immobilized [39] using external constraints. But this method makes the animal uncomfortable and hence the data collected is not fully ecologically valid; furthermore, such restraint is aversive and considered to be a higher level of invasiveness from an animal welfare point of view. The second approach is to independently record motion parameters during the scan, and then retrospectively use it to correct for motion or as a regressor of no interest in the activation analysis. This method is more ecologically valid, so in our experimental setting, we utilized an optical head motion tracking system [23], [40] based on a single camera to monitor and record motion parameters. However, originally this system was not specially designed for dog imaging, and thus can be adopted for general use. The advantages of single camera system over multi-camera systems are as follows [23]. First, the former does not need calibration of the angle and settings between the cameras, which must be routinely and repeatedly established in the daily function of the MRI facility. Second, the former avoids the technical difficulty of mounting multi-camera systems in-bore. Third, many MRI bores limit the field of view of cameras and this narrow aperture hinders the placement and efficacy of the second camera.

The single camera optical head motion tracking system was an MRRA Inc. model HT-1000 comprised of an IR (infrared) illuminator, an optical IR camera with a built-in DSP (digital signal processing) processor, a dot reflector, a video monitor and a palmtop computer. The IR illuminator provided an IR source that was reflected by the dot reflector mounted on the dog's head; the IR camera picked up the reflected IR light from the dot reflector and aided in calculating the change in dot position. Specifically, the image taken by the IR camera was binarized so that the round dot reflector was clearly separated from the background. The binarized image, the x, and y displacement of the centroid of the dot reflector as well as its area calculated by the DSP processor in the IR camera were digitally transmitted to the video monitor and palmtop computer. The sampling rate was 1 kHz. By doing so, we obtained the time series of the x, y coordinates and the area in units of millimeter and mm^2^, respectively. Then, these time series were downsampled to fMRI temporal resolution of TR (repetition time)  =  1 s, and the relative displacements of x(t), y(t) with respect to x(0), y(0), which were the x, y coordinates corresponding to the first fMRI volume, were obtained and used as a regressor of no interest (*Q_i_*(t) as in Eqn. 3 of “General Linear Modeling and Statistical Testing” section which is explained later) in the activation analysis, after correcting for motion using image transformation based realignment. The dot reflector was a one-inch diameter disk of engineering grade-10 retro-reflective tape (3M Corp.) attached by adhesive to the forehead of the dog. The video monitor allows the operator to check for proper image framing.

It is noteworthy that the system described above was only capable of 2-dimensional tracking, i.e. x and y directions, anterior-posterior direction missing. However, with a properly designed 3D target [23] and scanner interface, prospective online motion correction could potentially be performed with animals which cannot be trained to minimize head movement.

### Stimulation Paradigm

Each run consisted of 5 blocks of odor stimulation of 10 s duration, with a fixed resting (no odor) interval of 30 s between the end of one block and the start of the next, as illustrated in the fMRI block design sequence in Fig. 5. The order of low and high concentration runs were randomized for both awake and anesthetized dogs. The no-odor interval was thrice the duration of the odor stimulation interval in order to prevent the adaptation of the dog's olfactory system to the odorant. Previously it was shown that repetitive brief odorant pulses (≤10 s) can evoke activity in the rat olfactory bulb measurable through fMRI [41]. The choice of 10 s for odor stimulation and 30 s for baseline was motivated by previous studies showing that such a paradigm is effective for eliciting measurable neural response and at the same time, prevent habituation [42], [43]. In an fMRI analysis of the rat olfactory bulb, odorant stimulation for 4.8 minutes failed to show a reduction in activation [44]. Within a much longer stimulation (27.6 min) the activation declined, displaying habituation. Extra- and intracellular recordings of the main olfactory bulb of the rat support these results, showing just a small reduction in activation when a long stimulus of 50 s was given [45].

### Data Acquisition

T_2_*-weighted functional images were acquired using a single-shot gradient-recalled echo-planar imaging (EPI) [46] sequence for blood oxygenation level dependent (BOLD) contrast on a Siemens 3 Tesla Verio scanner. Two hundred temporal volume repetitions of 14 axial slices with 3 mm thickness were acquired using the following parameters: repetition time (TR) = 1000 ms, echo time (TE) = 29 ms, field of view (FOV) = 192×192 mm^2^, flip angle (FA) = 90 degree, in-plane resolution 3×3 mm, in-plane matrix 64×64, and whole brain coverage. A total of 34 low and 34 high concentration runs were obtained from anesthetized dogs whereas a total of 32 low and 32 high concentration runs were obtained from awake dogs. However, 4 runs of low and 4 runs of high concentration obtained from awake dogs were excluded due to excessive motion. The exclusion criterion was: >10 mm displacement between two consecutive acquisition time points in x, y or z direction. Also, in the z direction, if there was 10 mm total displacement between any two acquisition time points in one run, it meant that the dog's nose was not fully inserted in the mask at some time. This would have jeopardized the odorant effect at that time point. Therefore, any runs with > 10 mm total displacement between any two acquisition time points in the z direction were also discarded. Anatomical images were acquired using magnetization-prepared rapid gradient echo (MPRAGE) [47] sequence for overlay and localization, with parameters as: TR = 1550 ms, TE = 2.64 ms, voxel size: 0.792×0.792×1 mm^3^, FA = 9°, and in-plane matrix 192×192, FOV = 152×152 mm^2^, number of slices: 104.

### Image Processing

Image processing was performed using SPM8 [28] and consisted of slice timing correction, realignment to the first functional image, spatial normalization to a template, spatial smoothing, and general linear modeling (GLM) analysis. For spatial normalization, we adopted a customized strategy to deal with dog data as described below. For spatial smoothing, a Gaussian smoothing kernel with full width half maximum (FWHM) of 4×4×4 mm^3^ was employed. Considering that the dog's brain is smaller than that of humans, the FWHM employed by us was smaller than a FWHM of 8×8×8 mm^3^ usually employed with human data.

#### Spatial Normalization

**Figure 7:**
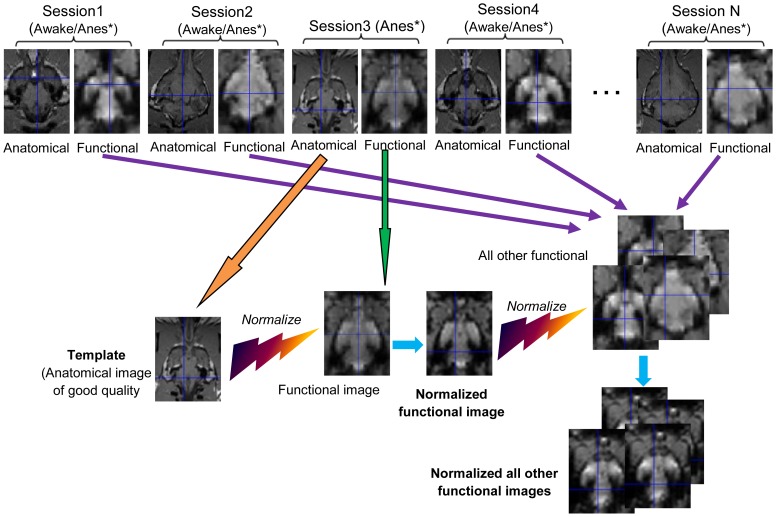
Flow chart of proposed spatial normalization procedure. A good quality anatomical image of an anesthetized dog was chosen as the template from Session 4 (4, being just an example). Then one functional image from the same session (Session 4) was chosen and normalized to the template. Subsequently, this transformed functional image was used as a template to normalize other functional images of other sessions. (*Anes:Anesthetized)

Spatial normalization [48] is necessary because dogs have different head shapes and sizes. Therefore, to perform group-level analyses, we need to normalize them to a standard template. For human as well as monkey and rodent imaging, a standard template is available (for example, the MNI (Montreal Neurological Institute) template for humans and MNI monkey atlas) [49]. Unlike human anatomical templates such as MNI which are derived using data from hundreds of subjects, the existing dog anatomical templates are derived from less than 10 dogs and hence do not capture the entire spectrum of head size variability [50]. Therefore, we adopted a more principled two-step approach. First, since for the normalization step to produce a good estimate of the spatial transformation, the modality of images should be similar, it was inappropriate to directly normalize all functional images of different dogs to one anatomical template. As a substitute, we chose a good quality anatomical image from the pool of anatomical images, obtained from an anesthetized dog, as the template. This choice was motivated by the fact that movement is negligible when dogs were anesthetized, and hence the quality of anatomical images obtained were superior. Then we chose one functional image from the same session as that of the anatomical template, and normalized it to the chosen template. Since both images were obtained from the same anesthetized dog in the same session, the normalization was relatively accurate and reliable. In the subsequent step, we used this transformed functional image as a template to normalize functional images obtained from other sessions (involving both anesthetized and conscious dogs). Since the images involved in this step were of the same modality, the normalization was relatively accurate. Note that by “session”, we mean the period during which we performed a number of runs for one dog. For example, in a session for one dog, we performed 1 structural run, 2 low concentration runs, 2 high concentration runs, and 2 resting state runs (the results from the resting state runs are not reported in this paper). So, one session included one subject with several consecutive runs in one day. The schematic of the proposed approach is shown in Fig. 7 and each step of the proposed normalization procedure was realized in SPM with the following parameters: 1. Template smoothing: 8 mm FWHM for step-1 and 4 mm FWHM for step-2. Since the structural image had higher resolution than the functional, we could afford to use higher smoothing in step-1 compared to step-2. 2. Source image smoothing: 4 mm FWHM for both steps. 3. Interpolation: 4^th^ B spline. 4. Voxel sizes: [2 2 2] mm.

### General Linear Modeling and Statistical Testing

#### Modeling the contrast of low/high odor concentration vs. rest condition

The pre-processed data was fed into a GLM for regression analysis and statistical testing of the effects of interest. The general linear equation can be briefly formulated as below.
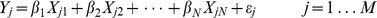
(1)


Where *Y_j_* is the response variable (fMRI time series at each voxel), *j* = 1…*M* indexes the observation; each *X_jk_* is an explanatory variable, *k* = 1…*N*, *β_k_*, *k* = 1…*N* are parameters to be regressed, under least square sense. In practice, the independent identically distributed (i.i.d.) normal distribution assumption for the error term is violated, but as long as we gather sufficient amount of data, the deviation from this assumption is deemed trivial. In our case, as indicated by the third sequence in Fig. 5, a block design for task conditions was employed, so a boxcar function corresponding to each of the two conditions (odor ON condition and odor OFF condition) was generated such that 
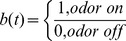
(2)


The boxcar function was then convolved with a standard hemodynamic response function (HRF) *hrf*(*t*) used in SPM so as to obtain a vectorial explanatory variable *X*_1_(t) = 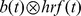. This HRF was not measured directly from dogs, but in order to model the variability of HRF in experimental data, we included time derivatives and dispersion derivatives of the HRF as regressors *X*_2_(t) and *X*_3_(t) in the GLM. Also, in order to regress out motion effects, the 6 rigid body transformation motion parameters *P_i_*(t), *i* = 1,…,6 resulting from realignment, and camera tracking parameters *Q_i_*(t), *i* = 1, 2 corresponding to head motion captured by the camera in *x* and *y* directions, were also added in the GLM as regressors. The resulting GLM equation is as follows.
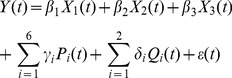
(3)


Where *β*, *γ*, *δ*, s are the coefficients and *Y*(*t*) is the observed fMRI signal at the given voxel. We tested for the statistical significance of *β*_1_, which represents the main contrast of odor versus rest, using a t-test and obtained corresponding activation maps.

#### Modeling the parametric modulation of the BOLD signal by low and high odor concentrations

We will use “parametric modulation” for short when referring to “parametric modulation of the BOLD signal by odor concentration” in the following sections. To test the effect of concentration modulation, parametric modulators were included in the GLM based on the following considerations. As is well known, the intensity of odor perception *I*, as a function of odorant concentration *C*, can be described by the Weber-Fechner law [17]: 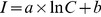
(4)


Where *I* is the perceived psychological intensity, *a* is the Weber-Fechner coefficient, and *b* is a constant. The experimental values of *a* and *b* measured for many odorants are approximately equal to 1.3 and 0.5, respectively. Thus, the change in perception due to a 10-fold increase in odorant concentration is equal to 1.3×ln(10)≈3 [12]. *In vitro* data recently reported by our group showed elegant agreement with this, i.e., a 10-fold increase in odorant concentration resulted in approximately 3-fold increase of the electro-olfactogram (EOG) signal [14], under the assumption that amplitudes of EOG signals elicited by various odorants *in vitro* correlate with perception of odor intensity. If we assume the fMRI signal has the same correlation with perception of odor intensity, a parametric modulation of the fMRI signal with a ratio of 1∶3 for increasing the odorant concentration by 10 times is suggested. Therefore, we concatenated one run of low concentration and one run of high concentration from the same session to form one observed variable. We constructed a parametric modulator *M* with a value of 1 for representing low concentration, and 3 for representing high concentration, and then included it into the GLM. So Eq.3 changes to Eq.5 as below.
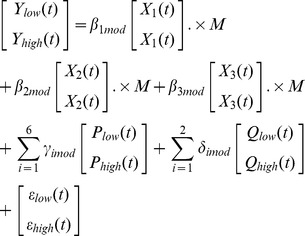
(5)


Here, .× means array multiplication, and subscripts “low” and “high” indicate corresponding regressors in Eq.5 obtained from low or high concentration runs, with corresponding coefficients named *β*_1mod_, *β*_2mod_, *β*_3mod_, *γ*_imod_, *i* = 1∶6, and *δ*_imod_, *i* = 1∶2.

After solving Eq.5 with regression coefficients as unknowns, a t-test was conducted for the significance of the parametric modulator *β*_1mod_. The numerical value of *β*_1mod_ represents the effect of the concentration modulation, meaning that the brain area with significant nonzero *β*_1mod_ showed three times the response for high concentration as opposed to low concentration.

#### Correction for Multiple Comparisons

To account for the false positive activations, we used a multiple comparison correction procedure called AlphaSim algorithm [51]–[53]. AlphaSim provides a means of estimating the overall significance level (the probability of a false detection) for an entire 3D functional image. This is accomplished by Monte Carlo simulation of the process of image generation, spatial correlation of voxels, voxel intensity thresholding, masking, and cluster identification. Based on the combination of individual voxel probability thresholding and minimum cluster size thresholding, the probability of a false positive detection per image is determined from the frequency count of cluster sizes. The underlying principle is that true regions of activation will tend to occur over contiguous voxels, whereas noise has much less of a tendency to form clusters of activated voxels. Therefore, the presence of clustering can be used as one criterion to distinguish between signal and noise. We used the dog brain mask created by ourselves as the image within which the activation was of interest to us and fed it into this algorithm. And we set the number of iterations to be 1000, the individual voxel probability threshold as p = 0.05, and consequently the minimum cluster size threshold was calculated to be equal to 15 voxels, corresponding to false positive detection probability  =  0.05 for the entire image.

## Results

### Spatial Normalization

**Figure 8:**
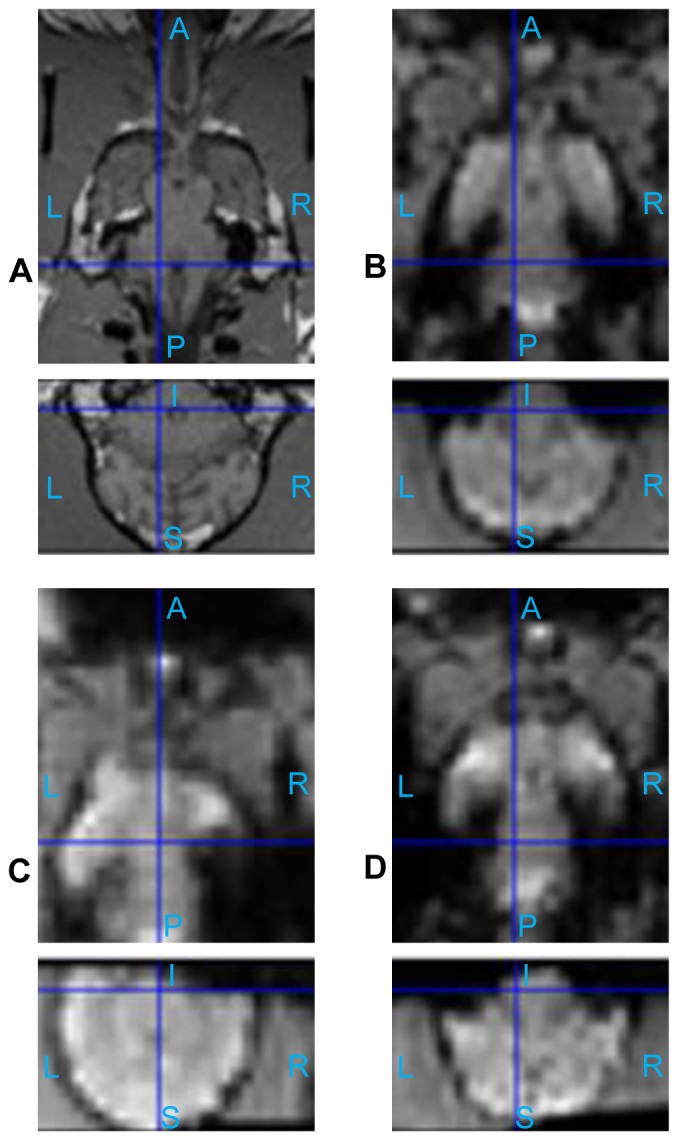
Normalization of functional images to anatomical template. The image (A) is the chosen anatomical template, its functional counterpart acquired in the same session is normalized to this template, which is shown in (B). The image (C) is a functional image of another dog. After normalization to the functional image (B), it becomes the image (D). (A: Anterior, P: posterior, S: superior, I: inferior, L: left, R: right)

Figure 8 shows one representative set of images before and after the spatial normalization using the procedure described in Figure 7. It can be seen that the functional image (Fig. 8(B)) is normalized well to its own anatomical (Fig. 8(A)). The mismatch between functional images from different dogs is conspicuous before normalization (Fig. 8(B) and (C)). After the second normalization step, they look similar (Fig. 8(B) and Fig. 8(D)).

### Low and High Odor Concentration in Anesthetized and Conscious Dogs

**Figure 9:**
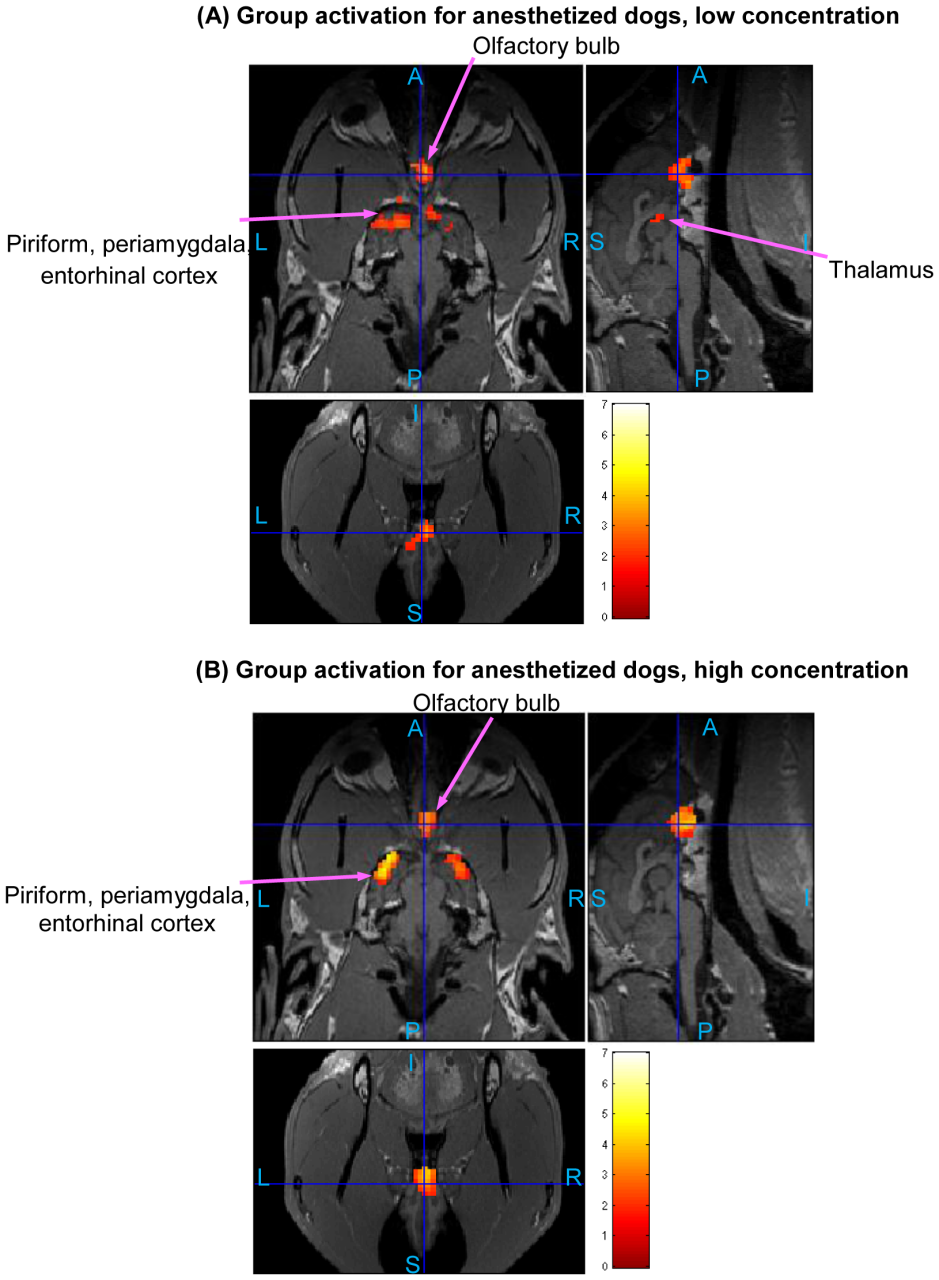
Group activation maps for anesthetized dogs. (Overall FDR = 0.05, cluster threshold  = 15 voxels using AlphaSim, t-contrast) Three orthogonal views are shown for each subfigure. Hot colormap is used for activation intensity, and important areas are indicated by arrows with labels. Subfigure (A) corresponds to low concentration odorant (0.016 mM), subfigure (B) corresponds to high concentration odorant (0.16 mM). (A: Anterior, P: posterior, S: superior, I: inferior, L: left, R: right)

**Figure 10:**
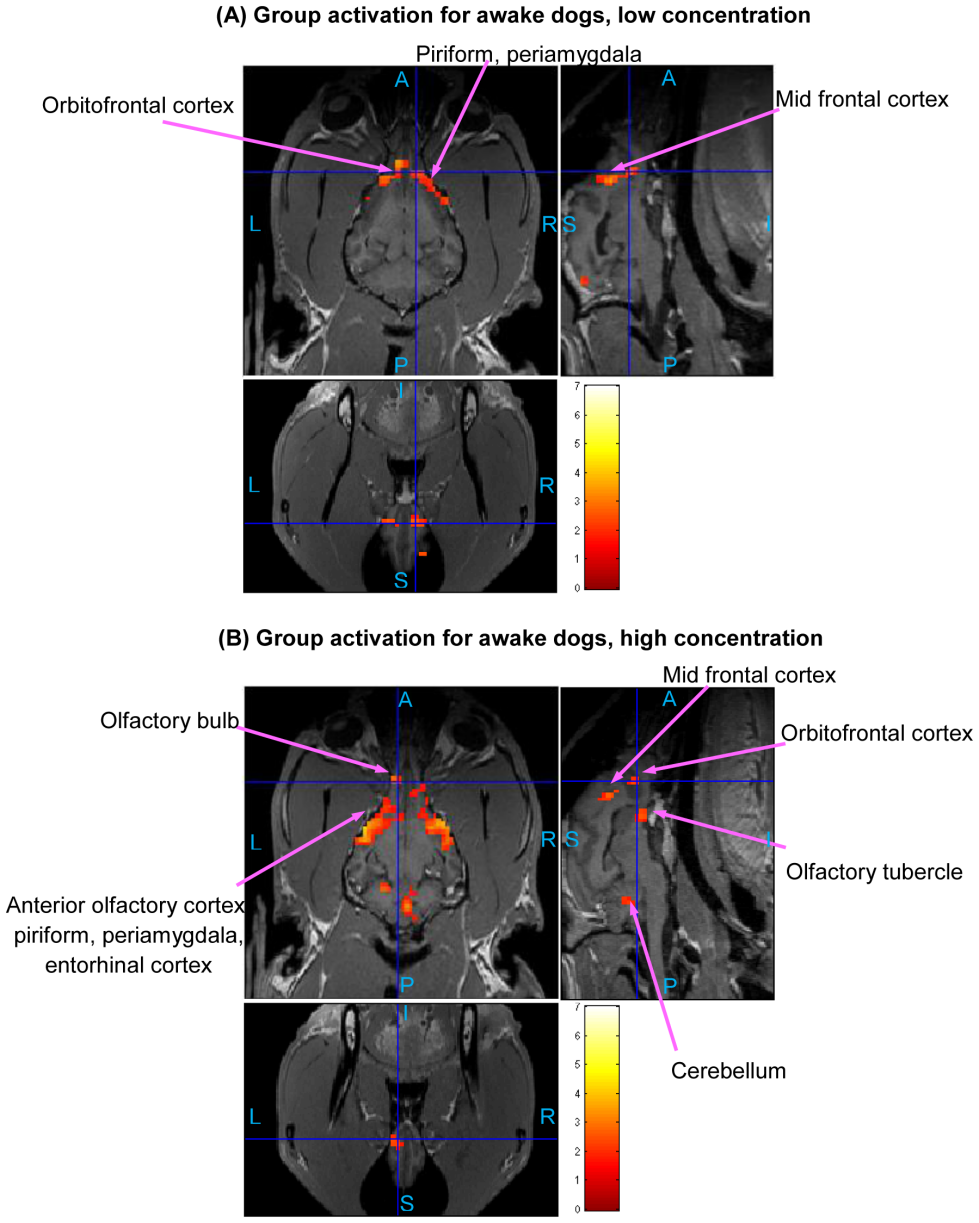
Group activation maps for awake dogs. (Overall FDR = 0.05, cluster threshold  = 15 voxels using AlphaSim, t-contrast) Three orthogonal views are shown for each subfigure. Hot colormap is used for activation intensity, and important areas are indicated by arrows with labels. Subfigure (A) corresponds to low concentration odorant (0.016 mM), subfigure (B) corresponds to high concentration odorant (0.16 mM). The activation in olfactory bulb for low concentration is not visible in this view, please refer to Table 3 for activation statistics with regard to this region. (A: Anterior, P: posterior, S: superior, I: inferior, L: left, R: right)

**Figure 11:**
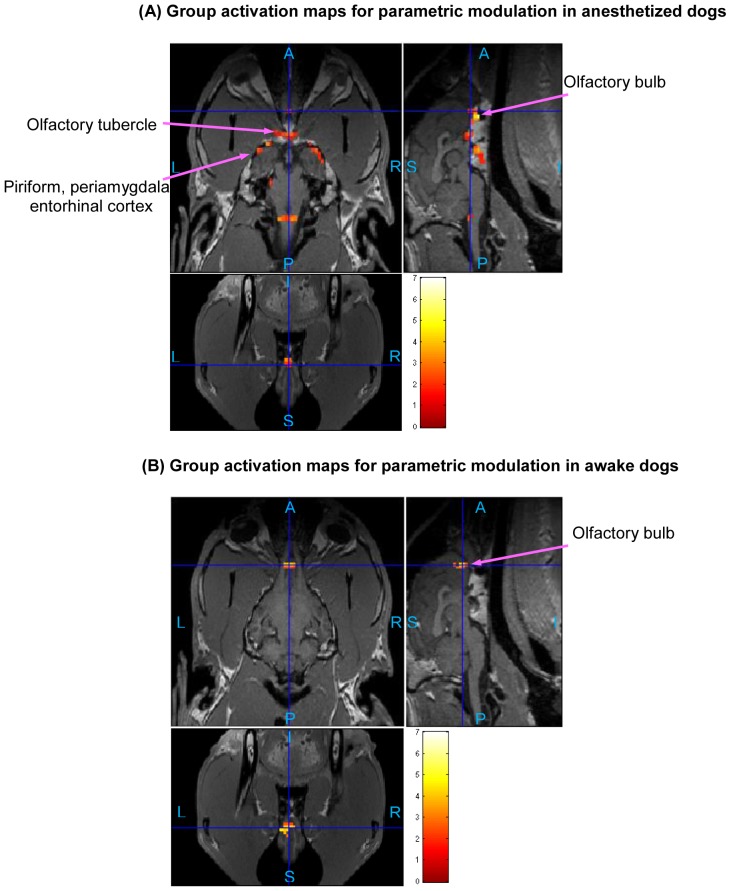
Group activation maps for parametric modulation in anesthetized dogs (A) and awake dogs (B). (Overall FDR = 0.05, cluster threshold  = 15 voxels using AlphaSim, t-contrast). Three orthogonal views are shown for each subfigure. Hot colormap is used for activation intensity, and important areas are indicated by arrows with labels. (A: Anterior, P: posterior, S: superior, I: inferior, L: left, R: right)

The statistical activation maps shown in Figs. 9, 10, and 11 were obtained using a t-contrast, cluster size threshold of 15 voxels calculated from AlphaSim algorithm applied to account for multiple comparisons, and individual voxel probability threshold p = 0.05 (the t threshold corresponding to this p value is 1.645). It is noteworthy that humans normally lie in the scanner facing up (i.e. head first-supine), but in our scenario, dogs were in “head first-prone” position in the scanner. Therefore, there is a difference in orientations between Figs. 8, 9, 10, and 11 in this paper and the routinely reported human images. The names of activated areas were identified by authors Edward Morrison and Vitaly Vodyanoy who have vast experience with dog neuroanatomy and olfactory physiology. They based their conclusions on visual comparison of activation images with published dog brain atlases [54]. Figure 9 shows activation maps obtained from anesthetized dogs for low odor concentration (Fig. 9A), and for high odor concentration (Fig. 9B). Corresponding cluster-level activation statistics are summarized in Table 1 and Table 2. Figure 10 illustrates the activation maps for low (Fig. 10A) and high odor concentration (Fig. 10B) in conscious dogs. Tables 3, 4 show corresponding cluster-level activation statistics for conscious dogs. For both awake and anesthetized dogs, we observed expected strong activation in the olfactory bulb and bilateral piriform lobes, including anterior olfactory cortex, piriform cortex, periamygdala, and entorhinal cortex for both low and high odor concentration. The ventroposterior location of the activation in the olfactory bulb is in agreement with previous studies in rats [55]. Visual comparison of the maps obtained from low odor vs. rest and high odor vs. rest contrasts reveals that the spatial extent and intensity of activations are larger for high odor concentration as compared to low concentration in both anesthetized (see Tables 1 and 2) and awake dogs (see Tables 3 and 4). A visual comparison of activation maps of anesthetized and conscious dogs shows dramatic differences in the spatial localization of activation. Activations in cognition-related areas such as medial, superior and orbital frontal cortices, and cerebellum are mainly found in conscious dogs.

**Table 1:** Cluster-level statistics of activations for anesthetized dogs, low concentration of odorant.*

Number of activated clusters: 4, total number of activated voxels: 365			
Cluster	Anatomical areas included	Number of activated voxels	Peak T value
#1	**olfactory bulb**	105	3.53
#2	**left anterior olfactory cortex, left piriform cortex, left periamygdaloid cortex, left entorhinal cortex**	146	3.05
#3	thalamus, hypothalamus	89	2.95
#4	**right anterior olfactory cortex, right piriform cortex, right periamygdaloid cortex**	25	2.64

*: ROIs shown in bold face were commonly activated for low and high (Table 2) odor concentration, as well as parametric modulation by odor concentration (Table 5).

**Table 2:** Cluster-level statistics of activations for anesthetized dogs, high concentration of odorant.*

Number of activated clusters: 5, total number of activated voxels: 424			
Cluster	Anatomical areas included	Number of activated voxels	Peak T value
#1	**left anterior olfactory cortex, left piriform cortex, left periamygdaloid cortex, left entorhinal cortex**, left frontal cortex	159	5.16
#2	**right anterior olfactory cortex, right piriform cortex, right periamygdaloid cortex**, right entorhinal cortex	97	4.16
#3	**olfactory bulb**	113	4.00
#4	**left piriform cortex**	24	2.75
#5	mid-cingulate	31	2.43

*: ROIs shown in bold face were commonly activated for low (Table 1) and high odor concentration, as well as parametric modulation by odor concentration (Table 5).

**Table 3:** Cluster-level statistics of activations for awake dogs, low concentration of odorant.*

Number of activated clusters: 3, total number of activated voxels: 379			
Cluster	Anatomical areas included	Number of activated voxels	Peak T value
#1	right superior frontal, right mid-frontal, right orbito-frontal cortex, right piriform cortex, right periamygdaloid cortex, right entorhinal cortex	124	4.15
#2	occipital cortex, **cerebellum**	172	3.75
#3	**Olfactory bulb**, left mid-frontal, left and central orbito-frontal cortex, left piriform cortex, left periamygdaloid cortex, left entorhinal cortex	83	3.35

*: ROIs shown in bold face were commonly activated for low and high (Table 4) odor concentration, as well as parametric modulation by odor concentration (Table 6).

**Table 4:** Cluster-level statistics of activations for awake dogs, high concentration of odorant.*

Number of activated clusters: 7, total number of activated voxels: 759			
Cluster	Anatomical areas included	Number of activated voxels	Peak T value
#1	bilateral anterior olfactory cortex, bilateral piriform cortex, bilateral periamygdaloid cortex, bilateral entorhinal cortex, medial and lateral olfactory stria, olfactory tubercle, left and right prefrontal, mid-frontal cortex	430	5.15
#2	**right cerebellum**	57	3.88
#3	**cerebellum**	185	3.64
#4	**olfactory bulb**	18	2.90
#5	left mid-frontal cortex	19	2.84
#6	left orbito-frontal cortex	27	2.81
#7	thalamus	23	2.58

*: ROIs shown in bold face were commonly activated for low (Table 3) and high odor concentration, as well as parametric modulation by odor concentration (Table 6).

### Parametric Modulation of BOLD signal by odor concentration

The differential response to odor concentration in terms of intensity can be quantified by using parametric modulators in GLM analysis as discussed before. The activated regions shown in Fig. 11 corresponding to anesthetized (Fig. 11A) and conscious dogs (Fig. 11B), respectively, have higher amplitude of activation in response to high as compared to low odorant concentration. Cluster-level activation statistics for parametric modulation are summarized for anesthetized and awake dogs in Tables 5 and 6, respectively. For anesthetized dogs, the regions showing parametric modulation are in the olfactory bulb, olfactory tubercle, piriform lobes, and part of brain stem, while those regions for conscious dogs are mainly in the olfactory bulb and cerebellum (see Tables 5 and 6).

**Table 5:** Cluster-level statistics of activations for parametric modulation of anesthetized dogs.*

Number of activated clusters: 8, total number of activated voxels: 345			
Cluster	Anatomical areas included	Number of activated voxels	Peak T value
#1	**olfactory bulb**	42	5.49
#2	medial and lateral olfactory stria, olfactory tubercle,	112	3.77
#3	right superior frontal cortex	35	3.49
#4	**right anterior olfactory cortex, right piriform cortex, right periamygdaloid cortex,** right entorhinal cortex	61	3.36
#5	brain stem	15	3.36
#6	**left anterior olfactory cortex, left piriform cortex, left periamygdaloid cortex, left entorhinal cortex**	26	3.26
#7	mid-occipital cortex	18	2.80
#8	left cerebellum	36	2.55

*: ROIs shown in bold face were commonly activated for low (Table 1) and high (Table 2) odor concentration, as well as parametric modulation by odor concentration.

**Table 6:** Cluster-level statistics of activations for parametric modulation of awake dogs.*

Number of activated clusters: 2, total number of activated voxels: 52			
Cluster	Anatomical areas included	Number of activated voxels	Peak T value
#1	**olfactory bulb**	24	4.53
#2	right occipital cortex, **cerebellum**	28	3.51

*: ROIs shown in bold face were commonly activated for low (Table 3) and high (Table 4) odor concentration, as well as parametric modulation by odor concentration.

**Figure 12:**
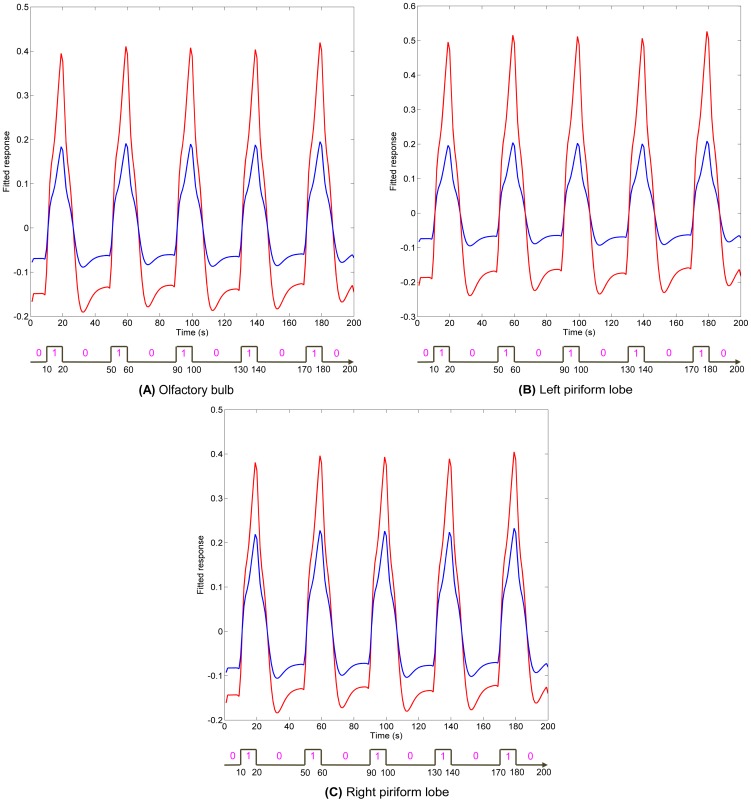
Comparisons of fitted time series obtained from the GLM for ROIs in anesthetized dogs. The ROIs are in brain regions that were activated by low and high odor concentration, as well as parametric modulation by odor intensity in anesthetized dogs. These regions are olfactory bulb and bilateral piriform lobes, which are shown in bold face in Tables 1, 2, and 5. In each of these regions, the ROI was determined by a sphere which centers at the peak activation of parametric modulation and has a radius of 2 mm. Fitted time series for low concentration are shown in blue, and high concentration in red.

**Figure 13:**
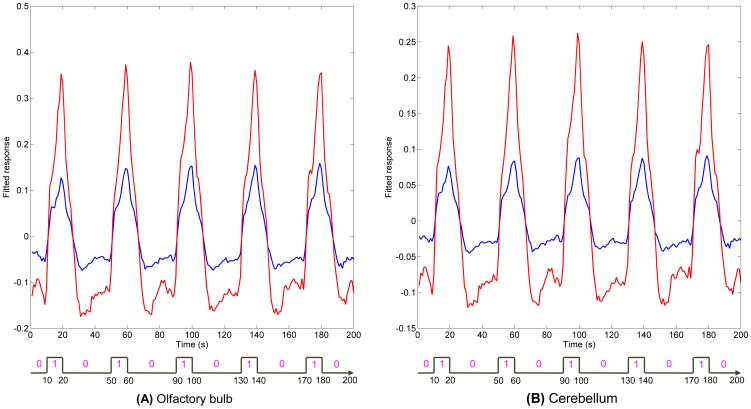
Comparisons of fitted time series obtained from the GLM for ROIs in awake dogs. The ROIs are in brain regions that were activated by low and high odor concentration, as well as parametric modulation by odor intensity in awake dogs. These regions are olfactory bulb and cerebellum, which are shown in bold face in Tables 3, 4, and 6. In each of these regions, the ROI was determined by a sphere which centers at the peak activation of parametric modulation and has a radius of 2 mm. Fitted time series for low concentration are shown in blue, and high concentration in red.

While Fig. 11 shows the spatial localization of regions parametrically modulated by odor concentration, Figs 12 and 13 show their temporal profile. Figure 12 gives a comparison of the fitted time series for low and high concentration in anesthetized dogs. The fitted time series were derived from the GLM and were mean time series for region of interests (ROIs) within anatomical areas which were activated by both low and high concentrations, as well as the parametric modulator. These anatomical areas were the olfactory bulb, left and right piriform lobes for anesthetized dogs. In each of the anatomical areas, the ROI was determined by a 2 mm radius sphere centered at the location of highest activation of parametric modulation. Fig. 13 gives a comparison of the fitted time series for low and high concentrations in conscious dogs. The anatomical areas were the olfactory bulb and cerebellum. These fitted time series demonstrate higher activation intensity for high, as compared to low, odor concentration, suggesting the modulation of response by odor intensity in these ROIs. It is noteworthy that the fitted time series for awake dogs in ROIs approximately follow the 1∶3 ratio in accordance with Weber's law [17] whereas in anesthetized dogs, it is approximately 1∶2.

### Effect of Motion

**Figure 14:**
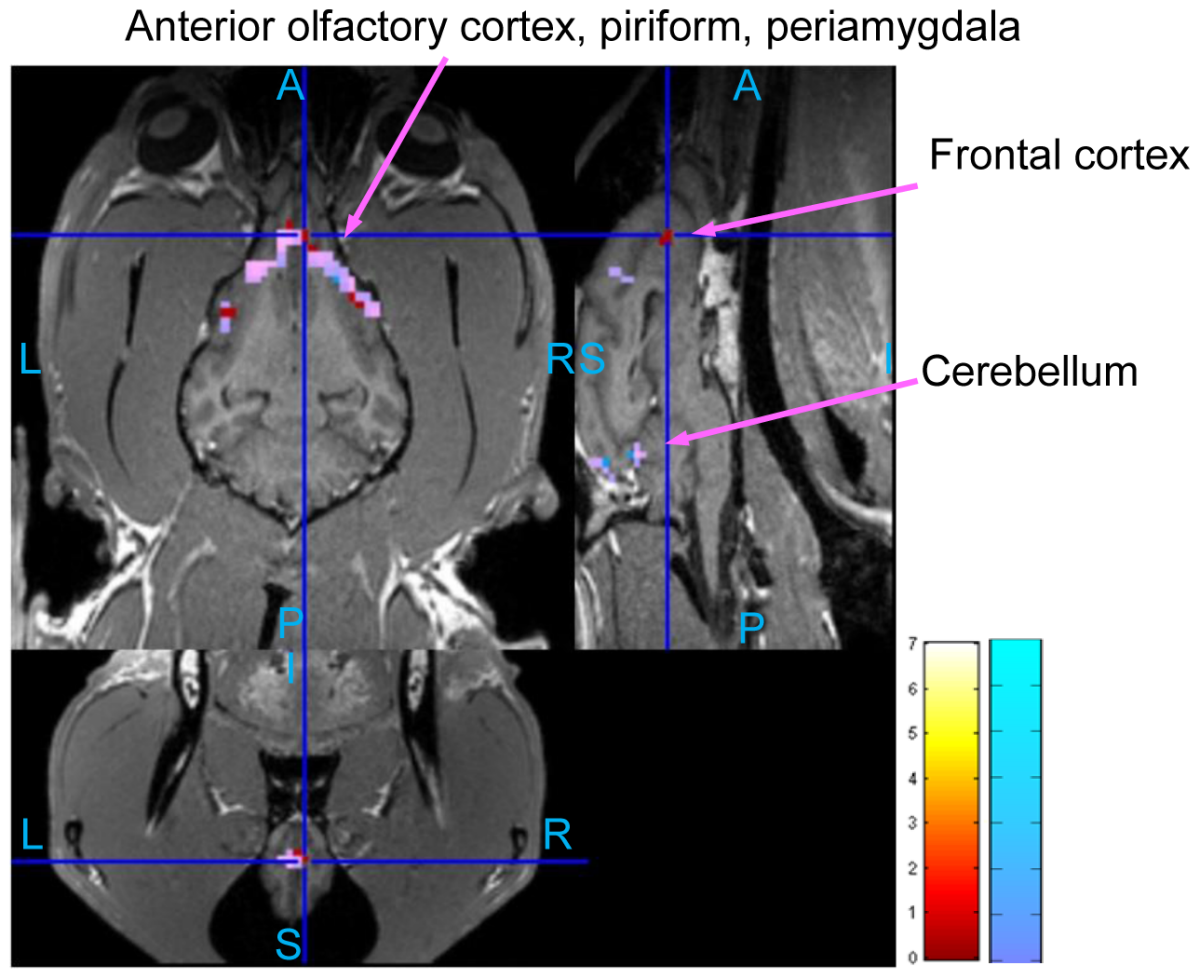
Comparison of activation maps with and without camera motion tracking parameters as regressors. (Overall FDR = 0.05, cluster threshold  = 15 voxels using AlphaSim, t-contrast) The activtion maps were for low concentration (0.016 mM) in awake dogs. The activation map obtained with only SPM realignment parameters as regressors is shown in cool colormap. The activation map with camera motion tracking parameters and SPM realignment parameters as regressors is shown in hot colormap. The common areas are overlaid such that they appear as purple. We found 3 clusters, 379 voxels in cool-colored map (same as in Table 3); 3 clusters, 396 voxels in hot-colorred map, and 3 clusters, 340 voxels in the common area. (A: Anterior, P: posterior, S: superior, I: inferior, L: left, R: right)

Figure 14 shows a comparison of the activation maps obtained with only realignment parameters obtained from rigid body transformations performed in SPM used as regressors in the GLM versus using both camera motion tracking and SPM realignment parameters as regressors in the GLM. It can be seen that, many activated areas can be identified using either methods, but there are non-trivial differences. We can see apparent additional activation in olfaction-related areas such as the orbitofrontal cortex and right piriform lobe when using motion parameters obtained from the camera as regressors in the GLM. This shows that jerky movements cannot be solely accounted for by SPM realignment and hence optical head motion tracking is valuable for imaging awake dogs.

**Figure 15:**
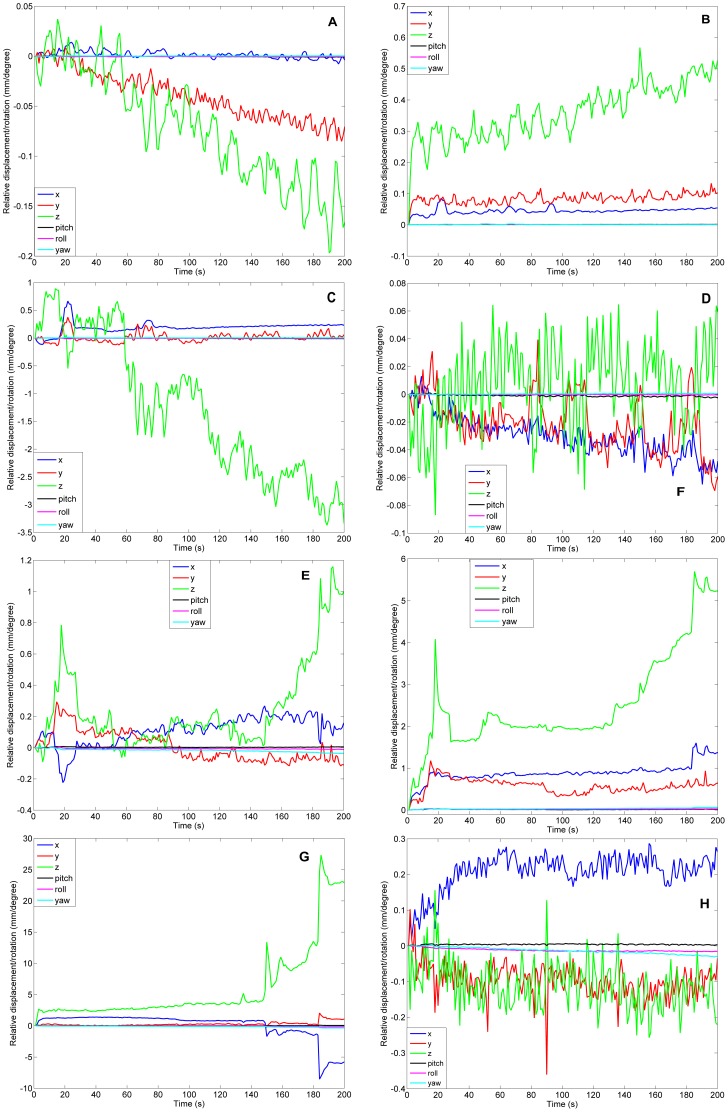
Affine parameters relative to the first functional image volume calculated by SPM realignment procedure. (A) mean time series of 6 affine parameters for anesthetized dogs; (B) standard deviation time series of 6 affine parameters for anesthetized dogs; (C) The time series of 6 affine parameters for the worst performing dog run at anesthetized state; (D) The time series of 6 affine parameters for the best performing dog run at anesthetized state; (E) mean time series of 6 affine parameters for awake dogs; (F) standard deviation time series of 6 affine parameters for awake dogs; (G) The time series of 6 affine parameters for the worst performing dog run at awake state (not included in the analysis); (H) The time series of 6 affine parameters for the best performing dog run at awake state.

Each subfigure in Figure 15 shows 6 realignment parameters obtained by using SPM for anesthetized or awake dogs. The realignment was referenced to the first functional image for each run, so all curves have zero values at the first time point, the basis point. Figures 15 (A) and (B) show mean and standard deviation time series of affine parameters for anesthetized dogs, respectively, while (E) and (F) show mean and standard deviation time series for awake dogs, respectively. (C) and (D) show affine parameters for the worst and best performing dogs, respectively, under anesthesia. Likewise, (G) and (H) show affine parameters for the worst and best performing awake dogs, respectively. Note that the worst performing awake dog was not included in the analysis. As expected, the motion for anesthetized dogs is significantly smaller, i.e. much smaller than the size of a single voxel, than that for awake dogs. Also, for awake dogs, we can observe jerky movements in Fig. 15. It is difficult to correct for such jerky movements using the realignment procedures based on rigid body transformations. Therefore, it is advisable to use optical head motion tracking to account for these jerky movements.

Additionally, we examined the proportion of variance in the signal explained by motion for the low/high concentration vs. rest condition contrast in awake dogs. For the proportion of signal variance explained by motion parameters in Eqn.3, we found the mean power (variance) of *δ_1_Q_1_*(t) to be 1.58 (since the fMRI signal does not have specific units, no units can be assigned to the power as well) and that of *δ_2_Q_2_*(t) to be 1.25. The power of *β_i_X*_i_(*t*), *i* = 1∶3 were 9.9, 4.6, and 2.6. Similarly, the power of *γ_i_P_i_*(t), *i* = 1 to 6, were 0.43, 0.35, 0.21, 0.18, 0.20, 0.31, wherein the first three values corresponded to x, y, z translations and last three corresponded to 3 rotations. So, the proportion of variance in the signal explained by camera motion parameters was (1.58+1.25)/(9.9+4.6+2.6+1.58+1.25+0.43+0.35+0.21+0.18+0.2+0.31) = 0.131, i.e. 13%, and the proportion of variance explained by SPM realignment parameters was (0.43+0.35+0.21+0.18+0.2+0.31)/(9.9+4.6+2.6+1.58+1.25+0.43+0.35+0.21+0.18+0.2+0.31) = 0.078, i.e. about 8%. Both were not very large. Hence this verified that retrospective motion correction sufficed.

## Discussion

Canine detection is one of the most efficient and effective tools used by law enforcement agencies to locate and identify a wide range of explosives and illicit substances. Despite the legendary accomplishment of the canine detector, a full understanding of its olfactory system is lacking. This study is the first to our knowledge to explore the canine olfactory system at *in vivo* cognitive level using fMRI. Previous studies explored the canine olfactory system either at *in vitro* cellular level, at the behavior level, or at the anatomical level. For example, at the cellular level, distribution of putative neurotransmitter amino acids in the dog olfactory bulb [9], anisole binding protein in dog olfactory epithelium [10] and changes of dog olfactory system with age [11] have been studied. Electrophysiological responses from the olfactory epithelium to odorant mixtures have been investigated to characterize the relationship between odorant stimulation and olfactory response at the cellular level [12]–[14]. Psychophysical studies regarding the dog's olfactory sensitivity [56], [57] and discrimination [58], [59], as well as behavioral assessments of trained detector dog performance characteristics have been performed [15], [16]. However, the neural substrates connecting the cellular level findings with behavior have been rarely explored. From our understanding of other sensory systems, the cognitive link is critical. For example, in the visual system, neurons in the primary visual cortex contain a faithful representation of the visual field [60]. However, the behavior of the organism is driven by different aspects in the visual field which are filtered through attention and higher level cognitive integration [61]. It is this contextual “understanding” of the visual field which drives behavior, rather than the entire visual field. By analogy, the olfactory map in the olfactory bulb is projected onto neocortical areas where an “understanding” of what the olfactory cue means to the organism at that moment is constructed, which drives behavior. Therefore, it is not possible to map cellular recordings in the olfactory bulb directly onto behavior unless a cognitive-level understanding is obtained.

This study is the first attempt to propose a framework to bridge this gap by performing noninvasive functional imaging of conscious and anesthetized dogs. We found activations in the olfactory bulb, anterior olfactory cortex, piriform cortex, periamygdala and entorhinal cortex for both awake and anesthetized dogs, and they were modulated by odor concentrations. The cerebellum, and cognition-related areas such as superior, medial, and orbital parts of the frontal cortex were activated mainly in the awake dogs. However, only the activations in the cerebellum and olfactory bulb were parametrically modulated by odor concentration in awake dogs. These activations are consistent with known anatomical projections from the olfactory cortex [62], [63]. It is noteworthy that mainly unilateral activations in low concentration condition in awake dogs became bilateral in the high concentration condition, in agreement with previous reports of such findings in human data [64]. Other structures, such as the hippocampus, which have structural connectivity with the olfactory cortex, did not have enough activated voxels to pass the cluster size threshold imposed by us for correcting for multiple comparisons. In addition, activation of higher order structures in conscious dogs is consistent with the role of homologous structures in other species (such as humans) as reported previously. For example, it has been shown in humans that the medial and orbital parts of the frontal cortex are involved in cognitive integration of all sensory stimuli in relation to prior experiences [6] and the cerebellum is implicated in sniffing and odorant threshold detection [65]. This shows that the olfactory stimulus is not being processed in higher cognitive structures in anesthetized dogs to the extent that it is in conscious dogs, thus justifying the need for awake dog imaging.

Our interpretation regarding the parametric modulation of the BOLD signal in 1∶3 ratio for a ratio of 1∶10 for odor concentration is based on the assumption that the scaling of EOG signal by odorant concentration can be extrapolated to the BOLD signal. Admittedly, the latter is not solely and linearly coupled with neural electrical activity. Rather, the latter is linked to neural activity through the convolution with HRF, or more accurately, the Volterra kernel of HRF [66]. Therefore, it is remarkable that we indeed used the same scaling with BOLD as with EOG to identify olfactory related areas which are parametrically modulated. However, there is need for cautious interpretation of these results till they can be replicated by other studies.

The utility of a practical method for assessing the cognitive processing of olfactory information in awake dogs is significant. Clinically, degradation of the sense of smell is a sentinel condition, particularly for neurodegenerative diseases such as Alzheimer's. The dog is recommended as a particularly good model of human age-related neurological disease as its brain shows neuropathologies and its behavior displays attendant cognitive deficits that are similar to that of humans [67]. Understanding normal cognitive processing of olfactory information may allow for identification of abnormalities of such processing well before it manifests in detectable alteration in sensory capabilities. Furthermore, the method may allow for elucidation of mechanisms underlying such olfactory degeneration that inform the etiology of such diseases.

The method described may allow for important enhancements in the use of dogs for detection of hazardous substances. The trained detector dog is widely regarded as the most versatile and capable tool for the detection of hazardous materials such as explosive devices [68]. However, there are many unresolved issues regarding how best to select, train, and employ dogs for such tasks [68]. The method described may allow for identification of olfactory processing characteristics of individual dogs indicative of exhibiting superlative performance in performing detection tasks. Thus, it may provide a means to select breeding stock for more efficient and effective production of working dogs. Description of the perceptual odor space (i.e., the physical dimensions responsible for the degree of similarity/difference of the perceptual experience of different odorants) through behavioral psychophysical methods has proven difficult and amenable to only relatively simple variation between individual chemical compounds [69]. Description of the perceptual space of olfaction relative to typical substances that are the targets of detector dogs, which are often composed of complex mixtures of chemical compounds, could significantly enhance detector dog technology. Detector dog technology could be greatly enhanced by the ability to examine the variation in cognitive processing of odorants that vary across different chemical dimensions, such as odorants to which a dog has been trained to detect vs. neutral odorants, through fMRI imaging of awake dogs. A cognitive processing model of the perceptual odor space could provide insights in explaining and ameliorating the occurrence of false alerts to particular non-target materials and misses of target materials within particular odor contexts. Phenomena such as the masking or overshadowing of target odors by other odors as well as the enhancement or disturbance of olfactory capability by the presence of particular substances could be examined using the awake fMRI imaging. Additionally, the ability to map cognitive olfactory processing could prove to be a valuable tool in characterizing the mechanism underlying the learning of odor discriminations. It is noteworthy that all of the dogs used in the current study had previous explosive odor detection training and their experience ranged from a few months of detector dog work in research projects to operational employment as a working detector dog. However, the odor detection experience of the dogs was not guided by any experimental design implications, rather, they were chosen because of their availability and because they are representative of the size and general disposition of dogs that are used as working detector dogs; hence allowing us to demonstrate the feasibility of this technique for future studies of such dogs. For the current study, none of the dogs had previous experience in detecting the specific odorants used in this study. We purposefully used odorant mixture with which the dogs had no learning history in order to not confound brain activity related to olfactory perception or the effects of previous learning history on perceptual processing of the odor stimulus. At the same time, the odorant mixture we have used is related to explosives and is recognized by dogs [70]. We intend to examine brain activity related to “learned” odors in future work by controlling the amount of exposure and training with specific odors, but in this first experiment, our intention was to confine our investigation to odors with which the dogs had no prior experience.

Apart from specific implications for the use of canine capabilities for detecting hazardous substances, our results also have implications for the advancement of dog non-invasive cognition and neurophysiology research in general. Previously researchers have made the case that physiological measures of canine cognition are required to complement behavioral studies, and have used non-invasive electroencephalography (EEG) to measure neural responses to auditory stimulus discrimination [71] and visual stimuli (specifically dog and human faces) [72], [73]. This work follows in their footsteps by incorporating fMRI as a non-invasive measure of brain function. Given the complementary strengths of EEG and fMRI, our work is likely to advance the cause of exploring the functions of deeper brain structures in canines. Further, the conclusions of this study have general implications for olfactory and sensory research in other animals.

In addition to the scientific contributions and implications discussed above, this work is likely to advance technical knowledge in the field of olfactory fMRI. First, head movement is a critical issue for fMRI. Excessive movement will corrupt the data and has been one of the barriers for imaging awake animals, though it is very well accepted that some cognitive processes are very difficult to study in anesthetized animals [20]–[22]. Specifically in the case of olfaction in dogs, we have demonstrated that different brain regions are recruited in awake and anesthetized dogs. In addition to passively studying cognition, even simple sensory acquisition may involve an active process which cannot be executed during anesthesia. For example, awake dogs can sniff but anesthetized dogs cannot, and hence jeopardizing the sensory acquisition process [74].

Though the case for awake animal imaging is strong, the technical challenges for achieving it are enormous. Some approaches used by previous researchers include the use of external constraints to limit motion [39] and head motion tracking using dual optical cameras in-bore [75] or external to the scanner [76]. Our proposed approach comprised of a combination of training and optical head motion tracking during awake dog imaging. The training part was employed to restrict large-scale movements which cannot be compensated retrospectively, while the optical head motion tracking part can account for jerky movements. In addition, our optical head tracking system was detached from the scanner, thus not suffering the problems caused by mount-in-bore camera systems. The results with integration of optical head tracking parameters showed the same basic activation pattern obtained by using only SPM realignment parameters, but also additional areas of activation in the orbitofrontal cortex and piriform lobe. This verifies the efficacy of the optical head tracking system. It is to be noted though, that constructing an appropriate target for the dog as done for humans before [23], could potentially allow the optical head motion system to track head movements in all six degrees of freedom thereby not requiring SPM realignment altogether. We will concentrate our future endeavors in this direction.

Next, the functional and anatomical data obtained from all dogs must be spatially normalized into one space for cross-subject comparison and group-level inference. We have proposed a strategy wherein we first chose one anatomical image with good image quality as a template, chose one functional image from the same session to normalize to the chosen anatomical, and the resulting functional image was then used as a template to normalize other functional images. This strategy was superior to directly normalizing all functional images to one anatomical template because, unlike human anatomical templates such as MNI which are derived using data from hundreds of subjects, the existing dog anatomical templates are derived from less than 10 dogs and hence do not capture the entire spectrum of head size variability [50].

Finally, the simultaneous delivery of odorants such that it is synchronized with fMRI data acquisition requires additional features in olfactometers. The most obvious one is that the components of the olfactometer which will be within the scanner's magnetic field and are involved in odorant delivery, be free of any metallic components. The desirable features of the device include computer control and odorant delivery of precise and reproducible duration at selected times, without any added stimulation (e. g., tactile, auditory) [30]. Our custom built device met all these specifications. Additionally, since it is portable, it can be used for both electrophysiological and fMRI studies.

## Conclusion

We have demonstrated a systematic method for performing functional magnetic resonance imaging of the olfactory system in conscious dogs. Specifically, we built a custom device for the delivery of olfactory stimulus and proposed a training procedure coupled with optical head motion tracking for imaging conscious dogs in an ecologically valid setting. Our results show many brain regions exhibiting odor concentration dependence. Importantly, higher order brain structures were activated mainly in conscious dogs, justifying the need for awake dog imaging. We hope that this seminal work will lead to further research in this area with implications for detector dog technology and national security.
